# Cancer Vaccines: State of the Art of the Computational Modeling Approaches

**DOI:** 10.1155/2013/106407

**Published:** 2012-12-23

**Authors:** Francesco Pappalardo, Ferdinando Chiacchio, Santo Motta

**Affiliations:** ^1^Dipartimento di Scienze del Farmaco, Università degli Studi di Catania, V.le A. Doria 6, 95125 Catania, Italy; ^2^Dipartimento di Ingegneria Elettrica Elettronica e Informatica, Università degli Studi di Catania, V.le A. Doria 6, 95125 Catania, Italy; ^3^Dipartimento di Matematica e Informatica, Università degli Studi di Catania, V.le A. Doria 6, 95125 Catania, Italy

## Abstract

Cancer vaccines are a real application of the extensive knowledge of immunology to the field of oncology. Tumors are dynamic complex systems in which several entities, events, and conditions interact among them resulting in growth, invasion, and metastases. The immune system includes many cells and molecules that cooperatively act to protect the host organism from foreign agents. Interactions between the immune system and the tumor mass include a huge number of biological factors. Testing of some cancer vaccine features, such as the best conditions for vaccine administration or the identification of candidate antigenic stimuli, can be very difficult or even impossible only through experiments with biological models simply because a high number of variables need to be considered at the same time. This is where computational models, and, to this extent, immunoinformatics, can prove handy as they have shown to be able to reproduce enough biological complexity to be of use in suggesting new experiments. Indeed, computational models can be used in addition to biological models. We now experience that biologists and medical doctors are progressively convinced that modeling can be of great help in understanding experimental results and planning new experiments. This will boost this research in the future.

## 1. Introduction

Vaccines for cancer represent an alternative approach to the use of standard drugs. Differently from the traditional vaccines that prevent disease instructing the immune system on how to recognize and destroy a particular pathogen, cancer vaccines enlist the patient's immune system to destroy existing cancer cells. While simple in concept, the development of products has proven difficult. Problems specifically lie in eliciting sufficient, tumor-selective stimulation of an immune system that is already tolerant of cancer cells [1, 2].

Revolutions in biotechnology and information technology have produced enormous amounts of data and are accelerating the extension of our knowledge of biological systems. These advances are changing the way biomedical research, development, and applications are done. Clinical data complement biological data, enabling detailed descriptions of various healthy and diseased states, progression, and responses to therapies. The availability of data representing various biological states, processes, and their time dependencies enable the study of biological systems at various levels of organization, from molecule to organism, and even population levels. 

Specific systems biology models, that is, applications of computer and mathematical models that enable the simulation of biological processes, can be used to investigate the physiology and pathology of the immune responses involved in vaccination and immunotherapy. This involves applications of computational simulations to the discovery, design, and optimization of vaccines and other immunotherapies. 

The term immunotherapies usually refers to the treatment of established disease while the term vaccine is restricted to prophylactic immune interventions. We will use “vaccines” to refer to generic immune intervention and will use terms “therapeutic vaccines” and “prophylactic vaccines” to distinguish between the two modalities. Vaccine design is amenable to the application of modeling techniques, for both the discovery and development of new and existing vaccines. In what follows, we first deal with a brief overview of different types of existing cancer vaccines; we then focus on modeling cancer vaccines and finally we draw our final remarks.

## 2. A Brief Overview of Cancer Vaccines

The ultimate aim of a vaccine is to activate a component of the immune system such as B lymphocytes, which produce antibodies or T lymphocytes, which directly kill tumor cells. Antibodies must recognize antigens in the native protein state on the cell's surface. Once bound, antibodies are capable of destroying tumor cells by means of antibody-dependent cellular cytotoxicity or complement-mediated cytotoxicity. T lymphocytes recognize proteins as major histocompatibility complex complexed with peptides that can vary in size, presented on the surface of the cells recognized. 

**Figure 1:**
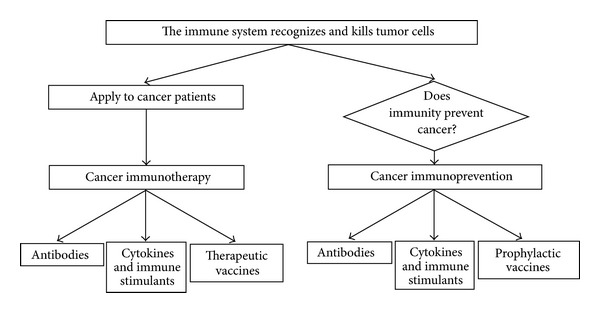
Tumor immunology and the main difference between cancer immunotherapy and cancer immunoprevention.

Recent research [3] demonstrated that the vaccine approach may also be useful in the prevention and treatment of cancer (tumor immunology, see Figure 1). It is known that the immune system eliminates most of the cancer cells (cancer immunoediting [4]). Those that are not recognized escape immune surveillance, leading to tumors. Tumor vaccines can thus be used to stimulate an immune response against poorly immunogenic tumor variants. In few words, the ultimate goal of tumor immunology is to understand the interactions between tumor and immune system cells and to devise immune based approaches to fight cancer.

The use of cytotoxic T cells (CTLs), dendritic cells (DC), and antibodies, actually represent well-known approaches in cancer immunotherapy [5].

The use of anti-idiotype (Id) antibodies as vaccines to stimulate immune system response against tumors, have been demonstrated effective in preventing tumor growth and curing mice with established tumors [6]. Several monoclonal anti-Id antibodies that have the appearance of distinct human tumor-associated antigens (TAAs) have been developed and tested in the clinic, demonstrating good results. Indeed the efficacy of these vaccines will depend on the results of several Phase III clinical trials. Numerous studies in mouse tumor models have shown that DCs pulsed with tumor antigens can induce protective and therapeutic anti-tumor immunity [7]. It is, however, worth to mention that the complexity of the DC system requires rational manipulation of DCs to achieve protective or therapeutic immunity.

Recently it has been shown that prophylactic vaccines administered to transgenic mice prone to cancer development can completely prevent tumor onset and restore a normal life expectancy [8]. Even though prophylactic cancer vaccines are still far from human application, this opens up an entirely new perspective in cancer prevention, leading to a future in which vaccines will equally contribute to the prevention of infectious diseases and cancer.

## 3. Modeling Cancer Vaccines

Computational models have been recognized as relevant for the understanding of biological systems. In particular, models are suitable for guiding biology from a qualitative to a quantitative, thus predictive, science. Pharmaceutical companies are starting to use models to optimize/predict therapeutic effects at the organism level, suggesting that computational biology can effectively play a key role in this field [9].

Obviously to model the behavior of a cancer vaccine, one needs to model the immune system that is one of the most exciting challenges as it represents one of the most complex biological systems. It is, in fact, an adaptive learning system that operates at multiple levels (molecules, cells, organs, organisms, and groups of organisms). Immunological research, both basic and applied, needs to deal with this complexity.

Computational immunologists increasingly use mathematical modeling and computer simulation to study the immune system and the immune responses to different pathogens [10]. Thus, quantitative models that appropriately capture the complexity (both in the architecture and the function) of the immune system are an integral component of the personalized medicine efforts. In silico models of the immune system can provide answers to a variety of questions, including understanding the general behavior of the immune system, the course of disease, the effects of treatments, the analysis of cellular and molecular interactions, and eventually the simulation of laboratory experiments. Here we will focus on modeling the immune response against tumors elicited by a cancer vaccine. 

**Figure 2:**
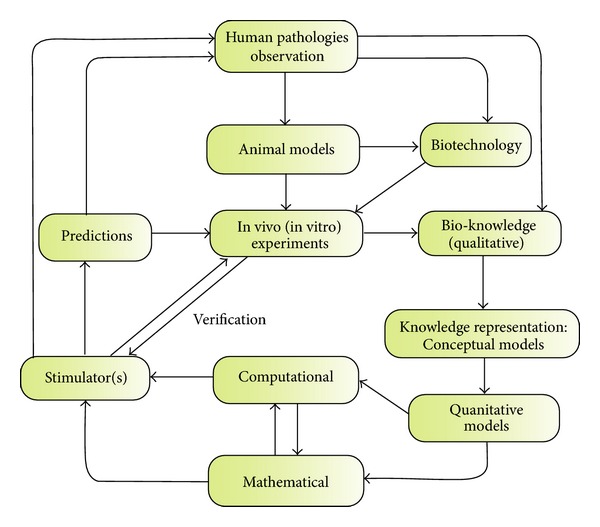
The modeling cycle.

Figure 2 summarizes the modeling cycle that all the modeling approaches should follow.

## 4. The SimTriplex Model

One of the first example in modeling a cancer vaccine is represented by the SimTriplex model. It is an agent-based model specifically tailored to simulate the effects of “Triplex” tumor-preventive cell vaccines in HER-2/neu transgenic mice prone to the development of mammary carcinoma [11, 12]. The Triplex vaccine blocks mammary carcinogenesis when administered to BALB-neuT mice starting at 6 weeks of age, allowing very long (>1 y) tumor-free survival [13]. The major limitation of the very effective Triplex vaccine was that only a Chronic protocol, with more than 60 vaccinations distributed throughout the life of the mouse, blocked mammary carcinogenesis, whereas shorter and/or delayed protocols left mice exposed to tumor onset.

SimTriplex includes a variety of cellular and molecular entities, including tumor and vaccine cells, B and plasma cells, helper and cytotoxic T cells, macrophages, dendritic cells, antigens, antibodies, and cytokines. The attributes of each cell entity include position, age, and state (e.g., resting, activated, memory, antigen-presenting, etc.). Changes of state (e.g., cell activation, cytotoxicity, cell death, etc.) are governed by a set of rules based on tumor immunology.

Antigen-specific immune interactions (antibody or immunoglobulin/antigen (IG/Ag) and T cell receptor/peptide/MHC (TCR-pMHC)) are modeled with bit-strings (sequences of 0 s and 1 s). Hamming distances is used as a measure of affinity among receptors and co-receptors: the probability of an interaction depends on the number of matches.

The simulation space is a two-dimensional triangular lattice (six neighbor sites) with periodic boundary conditions. Cells and molecules are free to move across the lattice sites. At each time step, representing 8 hours of real time, cells and molecules residing on the same lattice site can interact.

To model the continuous carcinogenic process of HER-2/neu transgenic mice, new tumor cells appear in the lattice, and existing tumor cells replicate (and rarely die). The simulation stops if the total number of tumor cells exceeds a threshold, signifying the formation of a palpable tumor mass, or after a defined number of time steps, typically more than 1 year of real time.

Probabilistic elements affect various starting variables (e.g., initial positions in the lattice) and interactions (e.g., cytotoxic death of tumor cells). The outcome of each run of the simulator, entailing the generation of a large number of pseudo-casual numbers, is taken to simulate the results of one mouse, thus reproducing experimental variability between individual mice. 

SimTriplex model coupled with optimization techniques (based on combinatorial optimization algorithms as genetic algorithms and simulated annealing) allowed to search for an optimal vaccination schedule to obtain the same efficacy of the Chronic protocol with a definitively reduced vaccine administrations. Simtriplex predictions has been verified in a in-vivo experiment (probably the first model results verified in vivo). Results show that in-silico predicted schedule does significantly reduce the tumors multiplicity on the ten mice mammary glands even if the vaccination efficacy for the first appearing of tumor was still overestimated. Further adjustment of the model is required to include evidence of immune aging which appeared from in vivo follow up results [13, 14].

## 5. The MetastaSim Model

The Triplex vaccine proved to be effective also as a therapeutic vaccine, showing its ability to be used against induced lung metastases [15]. Briefly, lung metastases were induced in BALB-neuT mice by intravenous injection of syngeneic mammary carcinoma cells.

The administration of the vaccine started one day after the intravenous injection of the metastatic cells and it is repeated twice weekly up to the end of the experiment (day 32), with lower but good prevention rates when the same cycle is started 7 days after the induction of the metastases (Triplex+7 protocol). The immunological responses in the immunoprevention and therapeutic experiments overlap only partially. A major goal of biologists is to better understand the biological behavior to improve the efficacy of the therapeutic treatment and to try to predict, for example, the outcomes of longer experiments in order to move faster towards clinical phase I trials. In a recent work [16], we developed a new computational model named MetastaSim to be used as an in silico virtual lab can help answering these questions. 

The MetastaSim model has been inspired by the SimTriplex model. MetastaSim has in common with SimTriplex the same modeling framework [17, 18] and some of the biological mechanisms shared by the in vivo experiments they model. However it has some important differences, that is, a complete revision of the cancer growth kinetics. The model is now able to simulate multiple different metastatic nodules, each one with its own growth rate, more accurately. To reproduce the growth in time of nodules, the Gompertz growth law is now used in its differential form. 

An exhaustive search for any optimal protocol has been performed. Results showed that it is possible to obtain in silico a reduction of approximately 45% in the number of vaccinations. Most of the protocols presented there share a similar vaccination strategy that is composed by a boost of three vaccine injections, a period of rest, and then a series of vaccine recalls that are somewhat equally spaced. The model suggests that any optimal protocol for preventing lung metastases formation should be therefore composed by an initial massive vaccine dosage followed by few vaccine recalls. Even if this is a well-known vaccination strategy in immunology, since it is commonly used for many infectious diseases such as tetanus and hepatitisB, it can be still considered a relevant result in the field of cancer-vaccines immunotherapy.

## 6. Model of Immunotherapy and Cancer Vaccination

Unfortunately, the efficacy of available therapeutic strategies for cancer still remain poor. Moreover, widely adopted approaches to cure or, at least, delay cancer development that is, chemotherapy and radiotherapy, both still carry major side effects for individual patients. In order to better understand therapies, experimentalists and clinicians are increasingly appreciating mathematical and computational modeling, and in recent years several papers appeared in the literature: they have begun to investigate the various aspects of the immune system response to cancer from a computational and mathematical perspective [19–21].

Particularly, in [22], the authors developed a mathematical model to describe the growth dynamics of an immunogenic tumor in the presence of an active immune response. They paid special attention on the interaction of cancer cells with cytotoxic T lymphocytes and professional antigen presenting cells in a relatively small, multicellular tumor, before the angiogenesis process. 

During the numerical simulation of the model, it has been discovered that adoptive immunotherapy protocols have the potential to promote tumor growth instead of inhibiting it. Conversely, active vaccination with tumor-antigen pulsed APCs was shown to be generally more effective.

## 7. Epitope Focused Immunoinformatics

DNA vaccination has been widely explored to develop new, alternative, and efficient vaccines for cancer immunotherapy. They offer several paybacks such as specific targeting, use of multiple genes to enhance immunity, and reduced risk compared to conventional vaccines.

Fast advances in molecular biology and immunoinformatics allow logical design methodologies. These technologies allow construction of DNA vaccines encoding selected tumor antigens together with molecules to direct and amplify the desired effector pathways, as well as highly targeted vaccines aimed at specific epitopes. Reliable predictions of immunogenic T cell epitope peptides are crucial for rational vaccine design and represent a key problem in immunoinformatics [23, 24]. 

For example, the authors in [25] explore the selection of T cell epitopes to develop epitope-based vaccines, the need for CD4+ T cell help for improved vaccines and the assessment of vaccine performance against tumor.

Moreover they present two applications, namely prediction of novel T cell epitopes and epitope enhancement by sequence modification, and combined rationale design with bioinformatics for creation of new synthetic mini-genes.

## 8. Repositories in Machine Learning Algorithms 

It is well known that the immune system is characterized by high combinatorial complexity, especially due to its wide potential repertoire. Consequently, the analysis of immunological data needs the use of specialized computational tools. A new way to select vaccine targets and reduce the number of necessary experiments is based on the use of machine learning (ML) algorithms in combination with classical experimentation. As the development of ML algorithms requires standardized data sets that are measured in a consistent way (and share the same uniform scale), there is a gap between the immunology community and the ML community. To overcome this problem and filling the gap, the authors in [26] present a repository for machine learning in immunology named Dana-Farber Repository for Machine Learning in Immunology (DFRMLI). Integrating experimental and in silico methods allows efficient study of highly combinatorial problems related with interpreting immune responses. With the advancement of experimental technologies, the amount of immunological data produced and distributed is increasing dramatically. Bioinformatics tools also based on statistical and ML algorithms are able to utilize these data. The main problem with both immunological data and other biological data is that they are usually represented qualitatively and as a consequence, these descriptions are often ambiguous, presenting a challenge for the mainstream ML developers. The DFRMLI is designed to overwhelm this hole through extending immunological data with well-defined annotations that could be conveniently used by the ML community. 

## 9. Models in Flow Cytometry Data for Cancer Vaccine Immune Monitoring

Detection of minimal residual disease, diagnosis, characterization of the profile of immunotherapies, and immune response tracking represent hot topics in cancer research. Flow cytometry (FCM) is widely used in these areas of interest. Circumventing spurious positive events and recognizing uncommon cells subsets delineate the challenge in all these applications. To accomplish this is task, the use of multiple markers simultaneously in the analysis of FCM data will help a lot. This because the additional information provided often lends a hand to minimize the number of false positive and false negative events, hence improving both sensitivity and specificity. 

With the use of the above explained strategy by manual gating, it is possible to analyze at most two markers in a single dot plot, often applying a sequential scheme. The sequential strategy is difficult to assess, as it gets rid of events that fall outside preceding gates at each stage.

Model-based analysis is a promising computational technique that works using information from all marker dimensions simultaneously and offers an alternative approach to flow analysis that can usefully complement manual gating in the design of optimal gating strategies. In [27], the authors presented results from model-based analysis illustrated with examples from FCM assays commonly used in cancer immunotherapy laboratories. 

The authors' approach to model-based analysis is based on the use of statistical mixture models. Statistical mixture models are very widely used in the presence of problems where objects depicted in several or many dimensions need to be clustered or classified. 

## 10. Modeling Personalized Response to Cancer Immunotherapy

Therapeutic interventions that stimulate tumor-specific immunity still remain rare. An improved understanding of patient-specific dynamic interactions of immunity and tumor progression, combined with personalized application of immune therapeutics, would increase the efficacy of immunotherapy. In [28] the authors developed a method to predict and enhance the individual response to immunotherapy by using personalized mathematical models. The approach is set in the early phase of treatment and includes an iterative real-time in-treatment evaluation of patient-specific parameters from the accruing clinical data, construction of personalized models and their validation, model-based simulation of subsequent response to ongoing therapy, and suggestion of potentially more effective patient-specific modified treatment. The model is then applied to a prostate cancer immunotherapy. The major finding of the simulations conducted in [28] suggested that an increase in vaccine dose and administration frequency would stabilize the disease in most patients. 

## 11. Immunotherapies Enhancing Vaccines

Recently, Wilson and Levy [29] have investigated the possible effect of an immunotherapy based on an immunoregulatory protein, the transforming growth factor beta, (TGF-*β*), in combination with vaccine treatments. The proposed mathematical model follows the dynamics of the tumor size, TGF-*β* concentration, activated cytotoxic effector cells, and regulatory T cells. Using numerical simulations and stability analysis, they have studied several scenarios: a control case of no treatment, anti-TGF-*β* treatment, vaccine treatment, and combined anti-TGF-*β* vaccine treatments. The model was able to capture experimental results, and hence has the potential to be used in designing future experiments involving this approach to immunotherapy.

## 12. Conclusions

The investigation of vaccines and therapeutic approaches against cancer from the mathematical and computational point of view is still a new field of research. It has been shown that several papers have begun to propose models that have been appreciated by both clinicians and experimentalists and have been proven to be of great use in improving anti-cancer approaches research. 

We expect that an extensive use of mathematical/computational modeling into clinical practice will stimulate the clinical research of new and alternative protocols for cancer treatments with immune interventions.

The possibility of the use of personalized approaches into the clinical practice is probably still far to come. However, virtual patient simulations can produce expected responses to the therapy for different class of patients (by immunological profile, age, pathologies, etc.). This can help the clinicians in deciding the best clinical approach for the specific patient. 

Finally, a thought on the future directions of the modeling cancer vaccines topic. We believe that models should be integrated during the entire cycle of cancer vaccine development line. This means that if a model has been used in the first line of the development (for example in the definition of epitopes targets), it should be used later in the optimization of the schedule and finally in the human response to the specific vaccine or immunotherapy. Presently, to the best of our knowledge, there is no model that has been applied to the three critical phases of vaccine development.
